# The extended analogy of extraembryonic development in insects and amniotes

**DOI:** 10.1098/rstb.2021.0268

**Published:** 2022-12-05

**Authors:** Kristen A. Panfilio, Susana M. Chuva de Sousa Lopes

**Affiliations:** ^1^ School of Life Sciences, University of Warwick, Coventry CV4 7AL, UK; ^2^ Department of Anatomy and Embryology, Leiden University Medical Center, 2333 ZC Leiden, The Netherlands; ^3^ Department for Reproductive Medicine, Ghent University Hospital, 9000 Ghent, Belgium

**Keywords:** extraembryonic development, insects, amniotes, amnion, serosa, developmental strategies

## Abstract

It is fascinating that the amnion and serosa/chorion, two extraembryonic (EE) tissues that are characteristic of the amniote vertebrates (mammals, birds and reptiles), have also independently evolved in insects. In this review, we offer the first detailed, macroevolutionary comparison of EE development and tissue biology across these animal groups. Some commonalities represent independent solutions to shared challenges for protecting the embryo (environmental assaults, risk of pathogens) and supporting its development, including clear links between cellular properties (e.g. polyploidy) and physiological function. Further parallels encompass developmental features such as the early segregation of the serosa/chorion compared to later, progressive differentiation of the amnion and formation of the amniotic cavity from serosal–amniotic folds as a widespread morphogenetic mode across species. We also discuss common developmental roles for orthologous transcription factors and BMP signalling in EE tissues of amniotes and insects, and between EE and cardiac tissues, supported by our exploration of new resources for global and tissue-specific gene expression. This highlights the degree to which general developmental principles and protective tissue features can be deduced from each of these animal groups, emphasizing the value of broad comparative studies to reveal subtle developmental strategies and answer questions that are common across species.

This article is part of the theme issue ‘Extraembryonic tissues: exploring concepts, definitions and functions across the animal kingdom’.

## 1. Extraembryonic tissues as a common strategy to the challenges of embryogenesis

**Figure 1.**
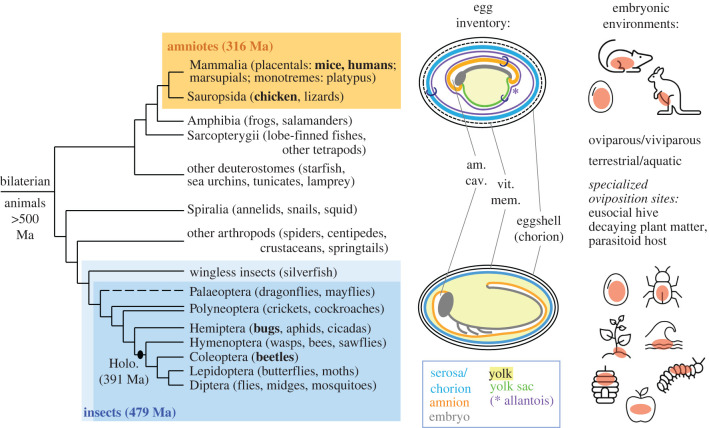
The phylogenetic and environmental context of animal EE tissues. Cladogram topology and divergence time estimates (left) are based on [1–5], with the dashed line and paired branches indicating weak monophyletic support or paraphyly, respectively. The primitively wingless insects are shaded in pale blue to indicate that while they possess proto-EE tissues, these never fully enclose the embryo (reviewed in [6]). The schematic egg diagrams (centre) are based on the chick and flour beetle. The small rings ('C') in the diagram of the chick embryo indicate that these mature tissues comprise contributions from two distinct germ layers: see below and figure 2 for developmental details for all five species in boldface type. The dashed line for the vertebrate vitelline membrane indicates its transient nature. The asterisk marks the location of the allantois (a waste sac and transient respiratory organ: not shown). The diversity of embryonic environments (right) is depicted graphically, with the location of the developing embryo in light red and the descriptors presented colinearly with the images (left-to-right and top-to-bottom, with the first three terms applicable to amniotes and all terms applicable to insects: see main text §§7 and 8); clip art images reproduced from Microsoft PowerPoint 2021, v. 16.52. Holo., Holometabola.

Embryogenesis is a period of extraordinary change. The fertilized zygote develops to generate all tissue types, and to correctly organize these in space and time to produce the correct morphological form and physiological function of a complete organism. This delicate period of the life cycle must be buffered from the external environment. There are two major and highly successful animal groups that have achieved this though the key innovation of extraembryonic (EE) tissues within the egg or womb (figure 1): the winged insects and the amniote vertebrates, comprising the mammals and sauropsids (reptiles and birds). As we review here, in each of these animal groups the EE tissues develop in parallel with the embryo proper, comprising some of the earliest tissue types to differentiate and mature. This enables them to play critical roles in protecting the embryo as well as directly fostering its development at mechanical, metabolic and genetic levels.

The EE tissues of insects and amniotes are evolutionarily independent, or analogous, as they were absent in the last common ancestor—an aquatic creature that arose over 500 million years ago (figure 1). That both crickets and chickens, and mosquitoes and mice, develop within a fluid-filled amniotic cavity represents a convergent solution to common challenges, including the demands of a fully terrestrial lifestyle. Adaptations of the egg to prevent desiccation, chiefly including the EE tissues, have enabled insects and amniotes to colonize diverse ecological niches away from the aquatic and humid habitats to which species such as amphibians and springtails are constrained [7,8].

Although named after its vertebrate counterpart, the insect amnion is evolutionarily older. The amniotic cavity is a defining trait of all winged insects [6], dating back to the Early Ordovician (479 Ma). Amniote vertebrates appear in the fossil record in the Carboniferous (316 Ma), after holometabolous insects—those with metamorphosis via a pupal stage, such as beetles, flies and butterflies (figure 1, and references therein). Insects are also far older when generation times are considered, which can be months in insects compared to years in vertebrates. Thus, the retention of EE tissues throughout winged insects is remarkable as an ancient trait. It is only in the past approximately 100 Ma, as holometabolous insects diversified in parallel with angiosperm radiation [3], that secondary loss of the amniotic cavity or an entire EE tissue occurred in restricted lineages of wasps and flies, including in the fruit fly *Drosophila melanogaster* [6,9]. Meanwhile, to the best of our knowledge, there have been no secondary losses of EE tissue in amniotes, although specific EE structures differ in prominence between species [10].

Here, we explore similarities in EE tissues and discuss biological features that govern the potential for species-specific variation. There are striking parallels in EE development between insects and amniotes, from genetic determinants to the morphogenetic basis of certain birth defects. However, a macroevolutionary comparison between these groups has been lacking. After a comparative account of EE development, with a focus on remodelling at tissue boundaries, we examine the genetic signature of the amnion. With the growing availability of stage- and tissue-specific atlases for gene expression, we document previously unrecognized commonalities that showcase avenues for future comparative investigation. We then consider morphogenetic and biomechanical properties of EE tissues, noting how EE development is intertwined with heart development and how genomic structure (polyploidy) underpins EE tissue functions. Finally, we conclude with a brief discussion of factors enabling EE diversification, distinguishing not only live birth (viviparous) and egg-laying (oviparous) gestation strategies but also the wider environmental context of embryogenesis.

## 2. Anatomical comparison of amnion and serosa/chorion between insects and amniotes

There are two EE tissues in both insects and amniotes (figure 1: ‘egg inventory’), albeit with a mix of semi-overlapping terminology to refer to different egg and EE structures. In both animal groups, the inner EE tissue is the **amnion**: it delimits a fluid-filled amniotic cavity that directly surrounds the embryo. The outer EE tissue, which differentiates first, has the primary role of mediating interactions with the outside world. In insects, the outer EE tissue is termed the **serosa**, and it is immediately subjacent to the eggshell, which is an acellular structure comprised of an outer chorion and inner vitelline membrane [11,12]. Not to be confused with the insect eggshell, the outer EE tissue in most amniotes is termed the **chorion** (or, traditionally in sauropsids, also the serosa [13]). In viviparous amniotes, it arises from the trophoblast (trophectoderm in human; EE ectoderm in mouse) and it will contribute to the placenta at the fetal–maternal interface, including in human and mouse [14,15]. In oviparous amniotes, the chorion derives from the EE ectoderm, and it develops to largely supplant a degenerating vitelline membrane, such as in the chick [13].

Similar to the vitelline membrane of oviparous amniotes, the zona pellucida of viviparous amniotes is a transient acellular surrounding layer, from which the blastocyst embryo hatches in very early development [16]. In contrast, the insect vitelline membrane is a permanent eggshell component that in fact crucially enables live imaging throughout embryogenesis, by offering transparency while maintaining egg structure (e.g. [17,18]).

Distinct from the amniotic cavity and perivitelline space between the serosa/chorion and eggshell, a third compartment is the yolk sac (visceral yolk sac in mice). While present in both insects and amniotes, this structure differs between species in two respects. First, for embryos that develop within an egg, the yolk sac contains lipid- and protein-rich yolk as nutrition for the developing embryo, whereas in viviparous amniotes the yolk sac content has a fluid-based composition [11,19,20]. Second, in amniotes the primitive endoderm (or hypoblast) extends beyond the embryo to constitute the EE endoderm as a tissue layer that surrounds the yolk [21]. In contrast, in insects the cortical structure of the yolk is termed yolk sac, but it is not a cellular layer in its own right [11,22,23].

**Figure 2.**
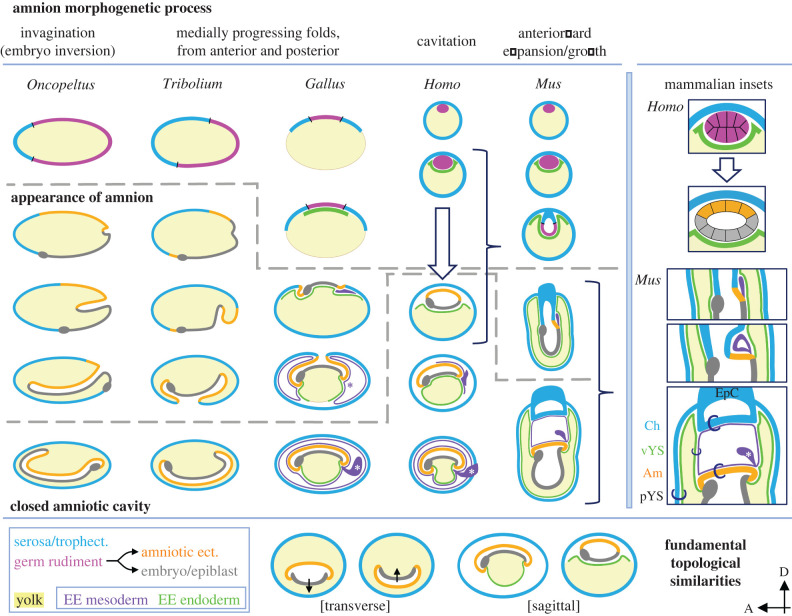
Comparison of early EE tissue differentiation and amnion morphogenesis in selected model species. Unless otherwise indicated, images are mid-sagittal views, with a grey oval indicating anterior of the embryo proper. Dashed lines demarcate the major events of the appearance of genetically and/or morphologically distinct amniotic ectoderm and amniotic cavity closure. For *Homo* and *Mus*, the curly brackets span stages shown in further detail in the inset images (right column). The colour scheme for tissue types is indicated in the legend (‘EE mesoderm’ and ‘EE endoderm’ are boxed, as these structures are amniote-specific). Tissue abbreviations: Am, amnion; Ch, chorion; ect., ectoderm; EpC: ectoplacental cone; pYS, parietal yolk sac (only in *Mus*); trophect., trophectoderm; vYS, visceral yolk sac. As in figure 1, rings in the final *Mus* inset image indicate the bilayered nature of amniote EE tissues. The purple asterisk indicates the site of initial outgrowth of the *Gallus* allantois. The white asterisk on purple tissue indicates the allantois/umbilical cord, comprised solely of EE mesoderm in *Mus* and of EE mesoderm and EE endoderm (not shown) in *Gallus* and *Homo*. Note that direct juxtaposition of serosal and embryonic tissue ventrally in *Oncopeltus* is supposition, pending the identification of early amniotic marker genes in this species. The *Gallus* embryo is not shown to scale relative to the yolk mass and enclosing EE tissues. Fundamental topological similarities are shown for the first four species (bottom row), with the insects in transverse aspect and with amniote EE mesoderm omitted for clarity. In insect transverse views, the arrow points to the dorsal side of the embryo, highlighting axial inversion of the embryo after invagination. Micrographs and previous schematics were consulted from multiple sources [13,14,16,24–27].

Amniotes also have an integral mesodermal contribution to the EE tissues that is without an insect equivalent. Differentiating from the epiblast (although in primates the EE mesoderm may arise from the hypoblast), the EE mesoderm expands to fully underlie all other EE tissue layers. It is when the EE membranes mature to an EE ectodermal–mesodermal bilayer that the monolayered amniotic ectoderm and trophectoderm become the bona fide amnion and chorion, respectively. Similarly, the yolk sac is an EE mesodermal–endodermal bilayer (distinct from the EE ectodermal–endodermal bilayer of the parietal yolk sac in mouse: figure 2, below). Thus, whereas the EE complement of amniotes integrates all three germ layers across the chorion, amnion and yolk sac, with each of these comprised of a bilayer, in insects the serosa and amnion persist as two simple (monolayer) epithelia of ectodermal origin. On the other hand, in some insects the serosa and amnion themselves adhere tightly in a bilayer to coordinate complementary morphogenetic functions in late development [18].

As amniote embryogenesis proceeds, metabolic demands of the growing embryo require further maturation of EE structures. Vascularization of the yolk sac metabolizes and transports yolk via primitive blood to the embryo proper [19,28]. In most amniotes, the EE mesodermal–endodermal allantois then stores waste products; in some eutherians (placental mammals) it contributes to the formation of a functional umbilical cord, while in sauropsids it transiently functions in respiration. In general, viviparous amniotes, where the embryo develops within the physiologically and structurally complex womb, show a pronounced reduction in the yolk sac and allantois compared with oviparous amniotes. Meanwhile, with their significantly smaller size (species-specific egg lengths of approximately 0.5–5.0 mm) and rapid embryogenesis (days to weeks), insects require neither feature. Insect yolk metabolism has been attributed to the serosa, amnion and persistent syncytial energids—nuclei with individual cytoplasmic islands but lacking cell membranes—that remain resident throughout the yolk mass, with catabolic products sequestered either within the amniotic cavity or perivitelline space [6,11,20].

## 3. Diverse strategies of early morphogenesis for extraembryonic tissue formation

Insects and amniotes are united by the possession of a serosa and amnion, which help to delimit the egg compartments. To form these structures and spaces, the predominant strategy is creation of the amniotic cavity from advancing serosal-amniotic folds. Yet within each of these two major animal groups, species employ different morphogenetic processes. To capture this commonality and some of the wider morphogenetic diversity of amnion formation, we compare five key species in detail (figure 2): the milkweed bug *Oncopeltus fasciatus*, the flour beetle *Tribolium castaneum*, the chicken (*Gallus gallus*), the human (*Homo sapiens*) and the mouse (*Mus musculus*).

**Figure 3.**
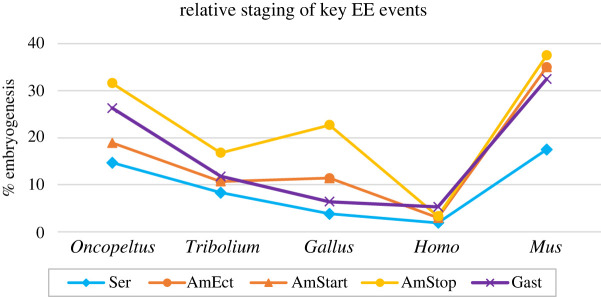
Relative staging of key EE events. Timing is shown as a percentage of total embryogenesis, graphically depicting the values for the five events detailed in table 1 (Ser: appearance of serosa; AmEct: appearance of amniotic ectoderm; AmStart: onset of amniotic cavity formation; AmStop: closure of the amniotic cavity; Gast: onset of gastrulation).

In oviparous species of insects and amniotes, early cleavage produces the blastoderm, an epithelialized cell layer on the yolk surface. Initial differentiation distinguishes the serosa from the germ rudiment, the latter comprising the presumptive amniotic ectoderm and embryo proper (figure 2: first row, first three species, table 1, figure 3). (For precision, we will use the vertebrate term ‘amniotic ectoderm’ for this monolayered ectodermal epithelium in both animal groups, while using either serosa or chorion for the outer EE tissue.) The amniotic ectoderm typically differentiates at the periphery between the serosa and embryo proper (figure 2: ‘appearance of amnion’). In most insects, amniotic cavity formation then initiates via apical constriction at the posterior egg pole (figure 2: insects' second row), with the bug and beetle representing the two predominant ways that this proceeds.

**Table 1.** Comparative timeline of key early events for formation of the amniotic cavity. Staging is given in absolute time (hours and days, as indicated) and in time relative to the total duration of embryogenesis (%, from fertilization to hatching/birth). The onset of gastrulation refers to the onset of internalization of embryonic mesoderm. This independent event is highly variable: across species, it occurs at three different times relative to the early events of EE development. The appearance/differentiation of the amniotic ectoderm is based on marker gene expression (not yet determined for *Oncopeltus*), which is generally concomitant with earliest cell shape changes for amniotic cavity formation. See also figures 2 and 3 for these events.

process/species	timing during embryonic development				
	*Oncopeltus* (at 25°C)	*Tribolium* (at 30°C)	*Gallus* (at 37°C)	*Homo*	*Mus*
appearance/differentiation of serosa/trophectoderm	28 h (14.7%)	6 h (8.3%)	20 h (3.8%)	5 d (1.9%)	3.5 d (17.5%)
onset of gastrulation, I.			34 h (6.4%)		6.5 d (32.5%)
appearance/differentiation of the amniotic ectoderm	unknown, pending marker genes	7.7 h (10.7%)	approx. 62 h (11.7%)	8 d (3.0%)	7 d (35%)
onset of morphogenesis for amniotic cavity formation	36 h (18.9%)	7.7 h (10.7%)	approx. 62 h (11.7%)	8 d (3.0%)	7 d (35%)
onset of gastrulation, II.	approx. 50 h (26.3%)	8.5 h (11.8%)			
closure of amniotic cavity	approx. 60 h (31.6%)	12.1 h (16.8%)	5 d (22.7%)	9 d (3.3%)	7.5 d (37.5%)
onset of gastrulation, III.				14 d (5.3%)	
total duration of embryogenesis	7.9 days	3 days	22 days	266 days	20 days
sources	[29–31]	[24,25,32]	[33–35]	[27]	[36]

In species like *Oncopeltus*, apical constriction leads to deep **invagination** of the amniotic ectoderm and embryo (figure 2: first column), with posteriorward serosal spreading maintaining tissue continuity over the yolk. Ultimately the lips of the invagination site close, sealing the serosa and the amniotic cavity [26,30]. A notable consequence of symmetric tissue invagination is that the embryo becomes inverted, with the head at the posterior egg pole and the ventral surface of the embryo facing towards the dorsal side of the egg. Embryo inversion during amnion formation occurs throughout the hemimetabolous winged insects (non-Holometabola), with morphogenetic reversal of this orientation in late embryogenesis—events that are collectively termed blastokinesis [6]. Amnion formation by invagination occurs throughout the Palaeoptera (dragonflies and mayflies), Paraneoptera (Hemiptera like *Oncopeltus* and close relatives such as thrips) and some species of beetle, moth and caddisfly (figure 1 [6]).

In contrast, in species like *Tribolium* the contiguous EE tissues envelop the embryo from advancing **folds** of internalizing amniotic ectoderm and spreading surface serosa, with the posterior amniotic fold particularly prominent in *Tribolium* (figure 2: second column). Ultimately, the medially progressing anterior and posterior folds join ventrally, involving intra-tissue fusion within each of the amniotic ectoderm and serosa concomitant with the separation of the two EE tissues [37,38]. Amnion formation from folds is predominant across the insects, including the many insect orders of the Polyneoptera and Holometabola (figure 1). Note that while the embryo maintains its orientation during amnion formation in *Tribolium* and in the Holometabola generally, embryo inversion also occurs during EE fold formation in some Polyneoptera [6].

Similarly, medially progressing EE **folds** envelop the chick embryo (figure 2: third column). Given the more extensive repertoire of EE tissues in vertebrates, folds of serosa-amniotic ectoderm advance in parallel with the development of the EE endoderm to envelop the yolk and of the EE mesoderm to underlie the other EE tissues and contribute to the allantois [13,16]. This method of amnion formation is typical of many amniotes, including sauropsids, marsupials, monotremes and some eutherians (ungulates and cetaceans, some carnivores, some rodents and rabbits) [39] and perhaps some cetaceans [40].

In the viviparous mammals, the formation of the amnion and chorion—and in general the implantation strategies in the maternal uterus—are notoriously diverse across species for both mechanism and timing [41–43]. In insects and amniotes with EE folding morphogenesis, closure of the serosa/chorion and closure of the amniotic cavity is a single event during or after gastrulation (table 1, figure 3). In contrast, in amniotes such as humans, the chorion and amnion form independently, with the former already established before the amniotic ectoderm differentiates. Then, early **cavitation** of the germ rudiment/inner cell mass is simultaneous with differentiation and epithelialization of the amniotic ectoderm and epiblast. Thus, the amniotic cavity is fully formed and sealed as the amniotic ectoderm arises, without an intermediate morphogenetic stage. It is only subsequently that the EE mesoderm forms (figure 2: fourth column and inset; table 1; [27]). Cavitation to produce the amniotic cavity occurs in some primates as well as some rodents and some bats [39].

Physical and temporal uncoupling occur in yet a different manner in the mouse (figure 2: fifth column and inset). There is early cavitation in this species, but this involves the trophectoderm and undifferentiated germ rudiment (presumptive amniotic ectoderm and embryonic epiblast). The amniotic ectoderm differentiates relatively late, after gastrulation begins, along the posterior side of the embryo. Its morphogenesis involves lateral and **anteriorward expansion**, accompanied by the EE mesoderm, to fuse over the head fold and thereby form the amniotic cavity [44].

Across species, the amniotic cavity is jointly delimited by the amnion and the embryo proper. However, this fluid-filled space is ventral to the insect embryo, while it is dorsal in amniotes. This may be a specific consequence of the general dorsal–ventral inversion of body organization between protostomes (including insects) and deuterostomes (including amniotes): in insects, the heart is dorsal, the digestive tract is medial and the nerve cord is ventral, whereas the converse is true in vertebrates [45]. Regardless, relative tissue topology is shared, with the amniotic cavity on the opposite side of the embryo to the yolk sac (figure 2: bottom row), ensuring that the region of the body where the gut will form has direct access to the nutritive yolk. Although fluid-filled, in many insects the amniotic cavity has a small volume. And, although mooted as a probable waste sac (§2), the composition of the insect amniotic fluid has yet to be characterized.

As noted above, aside from specific morphogenetic mechanism, there are some intriguing heterochronic differences in EE development between species. There is far greater temporal variation in the appearance of the amniotic ectoderm in vertebrates, whereas this is an early event in insects, both relatively and absolutely (figures 2 and 3, table 1). On the other hand, not only do insects lack EE endoderm, but the endoderm of the embryo proper is an extremely late derivative in insects, such that the embryo effectively only consists of two germ layers during amniotic cavity formation and the period generally thought of as gastrulation [20,46,47].

Lastly, the lineage of the amniotic ectoderm may differ across insects. In most species, the differentiating amniotic ectoderm has gene expression, cell shape and mitotic activity akin to its fellow germ rudiment derivative, the embryo proper, and distinct from the serosa [17,25,48]. This may differ in the Diptera, which exhibit reductions in amniotic tissue, loss of an amniotic cavity, conflation of the EE tissues into a single amnioserosa that only covers the yolk, and in extreme cases even stochastic, fatal loss of the amnioserosa altogether [9,23,49,50]. In some fly species, marker gene expression implies that the amnion arises at the periphery of a unified EE ectodermal territory [50], reassigning this tissue's lineage (discussed in [6]). On the other hand, dynamic gene expression spanning the serosa, amniotic ectoderm and embryonic ectoderm occurs widely in insects (e.g. [17,38,51]), highlighting outstanding questions about tissue-specific genetic signatures.

## 4. Deciphering the genetic signature of the amnion

It can be difficult to obtain amniote embryos in sufficient quantities at desired stages, as the embryos need to be manually dissected from inside the mother for all viviparous and the earliest oviparous embryonic stages. Also, oviparous embryos often require manual extraction from large eggs with opaque, hard shells. One of the interesting advantages of studying insects is that embryonic development takes place outside the mother's body and large numbers of embryos can be readily obtained. In many species, such as *Oncopeltus* and *Tribolium*, fertilization is concomitant with oviposition, providing access to all embryonic stages, and the eggshell is transparent or can be bleached. Combined with fast embryonic development and ease of performing genetic manipulations in insects, this has led to a large body of evidence regarding the gene regulatory networks that regulate the development of the serosa/chorion and, increasingly, the amnion.

In both insects and amniotes, by the onset of gastrulation there are multiple early genetic markers for the presumptive serosa/chorion. These include *Tc-zen1*, *Tc-zen2* and *Tc-hnt* for *Tribolium* [52] and *Cdx2*, *Elf5* and *Esrrb* in *Mus* [53,54] (table 2). Upstream regulation of the presumptive insect serosa requires a subset of axial patterning determinants for anterior and dorsal regions of the blastoderm (figure 2, e.g. [48,51]). Downstream, RNA-seq after RNAi and pathogen-challenge studies have identified factors for serosal tissue maturation and physiology [24,55,56].

**Table 2.** Selected orthologous genes in insect and amniote model species for developmental genetics. Gene names in boldface text are orthologues with EE expression and/or function (see main text). For lineage-specific duplications, paralogues may be collectively orthologous to other species' single-copy genes: these are listed in the same table row. Orthology determined based on the resources in box 1. Abbreviations: GPCR, G protein-coupled receptors; HD, homeodomain; TF: transcription factor; ZF: zinc finger.

molecular function	*Drosophila melanogaster*	*Tribolium castaneum*	*Gallus gallus*	*Homo sapiens*	*Mus musculus*
serosal expression					
TF (HD)	***Dm-zen (CG1046), Dm-z2 (CG1048)***	***Tc-zen1 (TC000921), Tc-zen2 (TC000922)***	*Gg-HOXA3/B3/D3*	*Hs-HOXA3/B3/D3*	*Mm-Hoxa3/b3/d3*
TF (C2H2 ZF)	***Dm-peb (CG12212)***	***Tc-hnt (TC009560)***	*Gg-RREB1*	*Hs-RREB1*	*Mm-Rreb1*
amniotic ectoderm and/or cardiac expression					
TF (GATA ZF)	***Dm-pnr (CG3978)***	***Tc-pnr (TC010407)***	*Gg-GATA4*	*Hs-GATA4*	*Mm-Gata4*
TF (HD)	*Dm-ara/caup (CG10571, CG10605)*	***Tc-iro (TC032451)***	*Gg-IRX4/6*	*Hs-IRX6*	*Mm-Irx4/6*
TF (T-box)	***Dm-Doc1/2/3 (CG5133, CG5187, CG5093)***	***Tc-Doc (TC012346)***	*Gg-TBX6*	*Hs-TBX6*	*Mm-Tbx6*
TF (other)	*Dm-TfAP-2 (CG7807)*	*Tc-AP2 (TC009922)*	***Gg-TFAP2A/2C***	***Hs-TFAP2A/2C***	***Mm-Tfap2a/2c***
TF (HD)	*Dm-Dll (CG3629)* (insects have a single *Dlx* homologue)	*Tc-Dll (TC009351)*	***Gg-DLX5***	***Hs-DLX5***	***Mm-Dlx5***
TF (GATA ZF)	***Dm-srp (CG3992)***	***Tc-srp (TC010405)***	***Gg-GATA1/2/3/6***	***Hs-GATA1/2/3/6***	***Mm-Gata1/2/3/6***
TF (HD)	***Dm-tup (CG10619)***	***Tc-tup (TC033536)***	***Gg-ISL1***	***Hs-ISL1***	***Mm-Isl1***
regulation of morphogenesis/cell shape (fog and GPCR signalling)					
secreted ligand	*Dm-fog (CG9559)*	***Tc-fog (TC006723)***	—	—	—
transmembrane receptor	*Dm-mthl1 (CG4521)*	***Tc-mist (TC010654)***	*Gg-GPR133, GPR144*	*Hs-ADGRD1, ADGRD2*	*Mm-Adgre5*
transmembrane receptor	*Dm-smog (CG31660)*	***Tc-smog (TC013504)***	*Gg-GPR158*	*Hs-GPR158*	*Mm-Gpr158*
G protein, alpha subunit	*Dm-cta (CG17678)*	***Tc-cta (TC034430)***	*Gg-GNA13*	*Hs-GNA13*	*Mm-Gna13*
structural protein, motor activity	***Dm-sqh (CG3595)***	***Tc-myosin II (TC030667)***	*Gg-MYL9*	***Hs-MYL9***	***Mm-Myl9, Myl12a***
transmembrane receptor (integrin)	***Dm-mys (CG1560)***	*Tc-mys (TC011707)*	*Gg-ITGB1*	***Hs- ITGB1***	***Mm-Itgb1***
FGF pathway featured components					
secreted ligand	*Dm-bnl (CG4608)*	*Tc-fgf (TC001760)*	*Gg-FGF20*	*Hs-FGF20*	*Mm-Fgf20*
secreted ligand	—	***Tc-fgf1 (TC034131)***	—	—	—
BMP pathway featured components					
secreted ligand	***Dm-dpp (CG9885)***	***Tc-dpp (TC008466)***	*Gg-BMP2/4*	*Hs-BMP2/4*	***Mm-Bmp2/4***
TF (MAD)	***Dm-mad (CG12399)***	***Tc-mad (TC033446)***	*Gg-SMAD1*	*Hs-SMAD1*	*Mm-Smad1*

Specific markers for the amnion have been difficult to identify, perhaps because this tissue emerges later in development and has a less pronounced genetic signature. In *Tribolium*, in contrast to *Mus* and *Gallus*, there are a number of amniotic markers, including *Tc-pnr* and *Tc-iro* [52,57]. However, these beetle genes are also expressed in embryonic tissues, and their respective vertebrate orthologues, *Gata4* and *Irx4/6* (table 2), are associated with early heart development in *Mus* [58,59] and *Gallus* [60,61] (see below), but not specifically with amnion formation.

In other cases, insect orthologues hold promise as a novel line of evidence in selecting new candidate genes for research into the amniote amnion (box 1). Transcriptomic datasets for the amnion have been generated for *Mus* [53,54] and *Homo* [74]. Moreover, single-cell RNA-seq datasets for gastrulating embryos of mouse [78,79] and human [75] are available. These datasets would benefit significantly from further exploration regarding the EE tissues, as they remain largely unexplored, with limited annotation and validation. Several open-source interactive platforms allow visual exploration of gene expression at the single-cell or tissue/organ level in *Homo* and *Mus* (box 1). From these, we have identified *TFAP2A/Tfap2a, TFAP2C/Tfap2c, DLX5/Dlx5* and *GATA3/Gata3* as markers of amniotic ectoderm in *Homo* and *Mus* as well as in *Gallus* [80,81]. However, these factors do not seem to cause a phenotype in the amniotic ectoderm when deleted in *Mus* [82–85], perhaps owing to redundancy with other family members. *DLX5* does not have a clear orthologue in *Tribolium* (table 2), and the expression of *Tc-AP2* (orthologue of *TFAP2A*) has not been investigated. However, the insect orthologue of *GATA3*, *srp*, has prominent expression in the *Tribolium* amnion [17] and the *Drosophila* amnioserosa [86,87], suggesting a notable degree of conservation in establishing amnion identity in both amniotes and insects.

Box 1. **Websites of interest to investigate amniote and insect genetics, genomics and gene expression in a comparative and regulatory network framework. Many of these sites are interconnected and with link-outs to wider genomic and protein classification sites.**

**Table:** 

description and citation	web link
multi-species integrated resources	
**Ensembl** is ‘a genome browser for vertebrate genomes that supports research in comparative genomics, evolution, sequence variation and transcriptional regulation’ [62].	https://www.ensembl.org/index.html
**Ensembl Metazoa** has genome information for over 100 non-vertebrate species, with a strong focus on insect pest species in VectorBase, including mosquitoes, sandflies and other flies [63].	http://metazoa.ensembl.org/index.html
**i5K Workspace@NAL** is the primary genome site for many insect and other arthropod species (over 90 species to date). Genomes and transcriptomes are BLAST-able, and community members can directly annotate gene models in Apollo, including for *Oncopeltus* and *Tribolium* [64].	https://i5k.nal.usda.gov
**STRING** **database** of protein–protein interactions documents billions of interactions based on diverse evidence types across thousands of species, including human, mouse, *Drosophila* and *Tribolium* [65].	https://string-db.org
**OrthoDB** provides evolutionary and functional annotation of proteins for thousands of species with sequenced genomes, including over 240 vertebrate and over 140 insect species. Orthology focuses on many taxonomic levels, with link-outs for InterPro, KEGG and others [66].	https://www.orthodb.org
species-specific resources	
**FlyBase** for *Drosophila* genes and genomes can be searched for integrated gene-level information, including isoforms, (mutant) alleles, phenotypes and also orthologues in other species [67].	http://flybase.org/
**BDGP** ***in situ* home page** documents gene expression throughout *Drosophila* embryogenesis, with controlled vocabulary for developmental anatomy. From the Berkeley *Drosophila* Genome Project (BDGP) [68].	https://insitu.fruitfly.org/cgi-bin/ex/insitu.pl
**iBeetle-Base** is a database of *Tribolium* RNAi phenotypes, integrated into gene pages with links to the genome browser, FlyBase homologues and OrthoDB (see above) [69,70].	https://ibeetle-base.uni-goettingen.de
**GEISHA** (*Gallus* Expression *in Situ* Hybridization Analysis) is the online repository of *in situ* hybridization and associated metadata for genes expressed during the first 6 days of chick embryogenesis [71].	http://geisha.arizona.edu/geisha/index.jsp
**GeneCards** is ‘a searchable, integrative database that provides comprehensive, user-friendly information on all annotated and predicted human genes’, and also function and orthologues [72].	https://www.genecards.org/
**MGI** (Mouse Genome Informatics) is ‘the international database resource for the laboratory mouse, providing integrated genetic, genomic and biological data’ [73].	http://www.informatics.jax.org/
***Homo* open-source interactive platforms** for visualization of gene expression at the single-cell or tissue/organ level: KeyGenes [74] and Human Gastrulation Data [75].	http://www.keygenes.nl http://www.human-gastrula.net/
***Mus* open-source interactive platforms** for visualization of gene expression at the single-cell level during and after mouse gastrulation [76–78].	https://marionilab.cruk.cam.ac.uk/MouseGastrulation2018/ https://tanaylab.weizmann.ac.il/embflow

Furthermore, changes in *GATA3* expression are associated with changes in BMP and FGF signalling in other vertebrate tissues [88,89], and both signalling pathways are required for correct amnion development in *Tribolium* [38,90]. BMP signalling has been shown to be functionally important for amnion development in *Mus* ([53,91,92], and see below), but not FGF signalling. This points to a common regulatory network (via GATA3 and BMP signalling) for amnion formation in insects and amniotes. Also, these comparative findings perhaps argue for further investigation of potential FGF signalling involvement in amnion development in other amniotes.

## 5. Morphogenetic and biomechanical requirements of the amnion throughout embryogenesis

The amnion needs to combine a high degree of elasticity with mechanical strength, first to accommodate its own morphogenesis during amniotic cavity formation and then to support the rapid growth of the embryo without rupturing, suggesting a set of unique biomechanical properties. In a third phase specific to insects, active withdrawal of the EE tissues in late development further places high mechanical demands on the integrity and remodelling capacity of monolayered, ectodermal epithelia.

In amniotes, where the amnion is an EE ectodermal–mesodermal bilayer, the mesoderm is critical for these properties. The amniotic mesoderm in *Homo* and *Mus* expresses high levels of *NRP1/Nrp1, POSTN/Postn, COL1A1/Col1a1, TAGLN/Tagln, ACTA2/Acta2* and *FN1/Fn1* [44,54,75], which are responsible for conferring both elasticity and strength. In *Mus* embryos defective for *Fn1,* gastrulation initiated and the knockout embryos formed EE mesoderm, showed a ‘closed’ amnion and chorion, and have an allantois, but the exocoelomic and amniotic cavities appeared to have defective pressure and distended shape [93]. In contrast, *Mus* embryos defective in *Foxf1* have defects in EE mesoderm and amniotic mesoderm expansion, resulting in the loss of elasticity [94].

In insects, a key factor for diverse early morphogenetic processes is *fog*, a secreted ligand that activates G-protein signalling to regulate myosin contractility and integrin activity. In addition to species-specific roles in the formation and integrity of the blastoderm epithelium and efficient internalization of embryonic mesoderm [17,95,96], Fog signalling is essential for EE morphogenesis in *Tribolium*. *Tc-fog* is required for initial apical constriction to drive amniotic fold formation and in the cuboidal-to-squamous cell shape transition for serosal spreading (figure 2: second and third stages depicted [17]). Across tissues and stages, in *Drosophila Dm-fog* is a regulator of *Dm-sqh* (*non-muscle myosin II* [95]), and both *Dm-sqh* and the integrin *Dm-mys* are required for late morphogenesis of the *Drosophila* amnioserosa [97–99]. Thus, although Fog signalling is an insect-specific innovation [17,96], it feeds into the regulation of fundamental components of cell shape maintenance and remodelling through G protein-coupled receptors (GPCRs), in particular through mechanoresponsive adhesion GPCRs (table 2; e.g. [100]). Using the open-source interactive platforms mentioned above, we report expression of the orthologues of *Dm-sqh* and *Dm-mys*, *MYL9/Myl9* and *ITGB1/Itgb1*, respectively (table 2), in EE mesoderm/mesenchyme in *Homo* and *Mus*.

As mentioned above, the highly conserved BMP pathway (table 2 [101]) is crucial for both patterning and early amnion morphogenesis in amniotes and insects. Disrupting BMP signalling in *Mus*, via genetic deletion of the ligand *Bmp2* or the cytoplasmic effector *Smad5*, resulted in defects in amnion/chorion closure, with subsequent malformations in heart development [53,91,92]. In both cases, the knockout mouse embryos developed until gastrulation, anteriorward expansion of the amnion/chorion occurred, and the EE mesoderm proliferated and created the exocoelomic cavity that lines other EE tissues and generates an allantois (figure 2 inset). However, by the time that the amniotic ectoderm and chorion EE ectoderm should detach from each other, giving rise to a closed amniotic cavity and ectoplacental cavity, this process failed, leaving an open proamniotic canal. This has severe consequences for further morphogenetic movements of the embryo, including pronounced cardiac defects. Similarly, impaired regulation of BMP signalling leads to delayed closure or a persistently open amniotic cavity in *Tribolium* [38].

As it matures, the insect amniotic ectoderm ceases mitosis and becomes polyploid (see below), yet its thinning must keep pace as the embryo rapidly doubles in length during germband extension [46]. This period of insect amnion development is poorly studied, in part because it often occurs deep in the yolk, but it offers fascinating remodelling challenges. For example, in *Oncopeltus*, the amnion tightly encloses each of the lengthening appendages (legs, mouthparts and antennae), giving it the character of a custom-fitted glove [26]. Yet, later the appendages fold medially and the amnion remodels to delimit a single, smoothly enlarged amniotic cavity. It would be intriguing to determine the cellular basis for such tissue structural plasticity, such as the relative roles of cell neighbour rearrangements or non-planar rotation [102].

Whereas in amniotes the EE tissues persist until birth/hatching, in most insects the EE tissues do not [6]. In mid-embryogenesis, the serosa and amnion dramatically end their lives by opening over the embryo's head, turning inside out as they peel back from the embryo, and compacting into a tissue mass that undergoes apoptosis within the yolk [18]. The later phases of EE withdrawal also occur in *Drosophila*, where contraction of the amnioserosa to the dorsal midline is required for dorsal closure of the embryonic epidermis: literally pulling the embryo's body together [103]. The tissues' mechanical properties are critical, with strong inter-tissue adhesion and precise timing of apoptosis [104]. The loss of EE tissue integrity (tearing) can leave constrictive belts of EE tissue encircling the insect embryo. This is strikingly similar to developmental defects in amniotes known as amniotic band syndrome or the ADAM complex (amniotic deformities, adhesions and mutilations), where the amnion fractures or tears [105,106]. In *Tribolium*, these defects can be genetically induced and investigated with high-throughput and high-resolution live imaging [18,38], offering an accessible research model to explore the link between early tissue mechanical properties and potentially stochastic outcomes.

## 6. Parallels in gene regulation and tissue properties in amnion and heart

We have noticed that some genes that are specifically expressed in the amniotic ectoderm in both insects and amniotes later become re-expressed in the heart, where they play direct roles in cardiac development. This is particularly intriguing given the tissues’ diverse topologies: whereas in amniotes the presumptive heart and amnion form an embryonic–EE boundary at the anterior of the embryonic disc, in insects the heart forms later and is not in contact with EE tissue [32,104]. For example, *ISL1* is required in cardiac progenitors in *Homo* [107], and it was recently reported to be expressed in the amniotic ectoderm in *Homo* and other primates [108]. Similarly*,* the *Drosophila* orthologue of *ISL1*, *Dm-tup*, is required in cardiac progenitors [109]. This is in addition to an EE-specific role of *Dm-tup* in maintaining amnioserosal integrity, which profoundly affects embryo body posture and thus, secondarily, the geometry of the developing cardioblast cell row [110]. This latter phenotype also occurs in *Tribolium* after knockdown of *Tc-Doc*, which has persistent amniotic expression [38].

In *Tribolium*, several amniotic marker genes are in fact also expressed in either mesodermal precursor tissue or in the cardioblasts themselves: *Tc-iro*, *Tc-Doc* and *Tc-pnr*. Whereas a cardiac role of *Tc-iro* has not been investigated and *Tc-Doc* knockdown does not produce an obvious primary heart defect, knockdown of *Tc-pnr* severely affects cardiogenesis, with the loss of cardioblast cells and substantial defects during heart tube formation [38,111,112].

Amniote orthologues of these dual amniotic/cardiac marker genes in insects vary in expression and function. The orthologue of *Tc-Doc, Tbx6*, does not have a prominent role in amnion or cardiac function, but rather functions in specification of paraxial mesoderm and the formation of the somites in both mouse and chick [113–115]. However, other members of the TBX family do contribute to heart development. This includes *Tbx5*, which shows a high degree of overlap with *Isl1*, as well as *IRX4/Irx4*, the vertebrate orthologue of *Tc-iro*, in the ventricular myocardium in *Mus* [59] and *Gallus* [60]. In *Gallus*, single-cell transcriptomics recently clarified that *IRX4* marks ventricular cells while *TBX5* specifically marks the left ventricle [116]. IRX4 seems to regulate heart chamber identity by regulating myosin and therefore contractile characteristics of the ventricular myocardium. Meanwhile, the *Mus* orthologue of *Tc-pnr, Gata4*, is an important regulator of early cardiac morphogenetic events, including tube formation and subsequent heart folding, rather than having a major role in cardiac mesoderm specification [58,117]. This function is conserved in chicken [118]. However, most probably there is redundancy between *Gata4* and *Gata6*, making it difficult to functionally separate the two.

A degree of similarity in the genetic networks in the two tissues (cardiac primordia and amnion) could be due to the biomechanical properties of the cardiac cell layer during folding, which requires elasticity with strength. But the similarity does not end there. The amniochorion in sauropsids shows spontaneous and rhythmic contractions, in particular after amniochorion closure (peaking at day 9 in the chick, with approximately 15 contractions min^−1^) [119,120], and this may explain its smooth muscle-like functionality. In *Mus*, the amniotic mesoderm clearly presents a smooth muscle-like genetic signature (*Acta2*+, *Tagln*+, *Myl9*+, *Tpm1*+ and *Cnn1*+). Due to limitations in culturing and live imaging a peri-implantation mouse embryo, contractile activity has so far not been described. However, in an *in vitro* model of amniotic injury in both *Mus* and *Homo*, amniotic cells with contractile characteristics are present at the wound edge [121]. Despite the very different structure of the squamous amniotic ectoderm in insects, pulsatile and peristaltic rhythmic behaviour in this tissue occurs during germband extension and dorsal closure [26,38]. Even if this originates in embryonic tissues, the insect amnion sustains and propagates these behaviours. Hence, it is perhaps not surprising to observe similarities in the molecular signature between the amnion and the heart, and it is remarkable that also in this regard there are clear parallels between amniotes and insects.

## 7. Polyploid genomic architectures underpin extraembryonic tissue functions

Tissues that support embryogenesis—both maternal and EE—often become polyploid, with multiple copies of the genome per cell instead of the typical diploid state. There is a growing body of evidence on how it is not only gene expression but also genomic architecture that underpins regulatory, physiological and protective tissue functions.

There are two notable polyploid EE tissues in placental mammals, each deriving from the trophectoderm via a distinct mode of polyploidization. Syncytiotrophoblasts develop by cell–cell fusion to become multinucleate, with discrete nuclei in a syncytial cytoplasm. This tissue is critical at the fetal–maternal interface, where it supports nutrient and gas exchange. It also helps maintain pregnancy by the secretion of placental hormones such as progesterone [122] and by immunological modulation to support maternal tolerance [123]. This large syncytium also serves as a protective barrier for the fetus, by virtue of its mechanically robust cytoskeletal meshwork and absence of intercellular junctions, which are susceptible to inflammatory responses and pathogen entry [123].

The fusogenic properties of syncytiotrophoblasts derive from domestication of genes acquired from retroviruses [124]. Exaptation of so-called *syncytin* genes occurred repeatedly in mammals, such that the genetic basis of placentation represents multiple instances of convergent evolution [124]. Intriguingly, marsupials have functionally equivalent viral-origin genes [125], although in most species the placenta is only a transient and relatively inefficient structure that precedes post-partum development within the marsupial pouch (it is known as the yolk sac placenta, or choriovitelline placenta, in contrast to the chorioallantoic placenta in eutherians [10,15]). Thus, the multinucleate character may be a byproduct of virally-derived invasive competence of the EE tissue.

In contrast, murine trophoblast giant cells (TGCs) become highly polyploid through endoreplication, generating up to 900 copies of the genome through DNA replication in the absence of cytokinesis [126]. This alternative mechanism of polyploidy also fosters strategies for physical protection and endocrinological support. As cell size is proportional to nuclear size, TGCs' ploidy may directly support tissue integrity and epithelial barrier function [127]. Moreover, increased DNA content need not be uniform. TGCs exhibit selective amplification of functionally important gene loci, such as for immune and hormonal regulation to support fetal physiology [128]. Similarly, polyploidy of maternal tissues in the *Drosophila* ovary is thought to support a high transcriptional yield of needed protein products for oocyte provisioning and eggshell production [127].

Endoreplication is also a hallmark of both the serosa and amnion in insects, with tissue-specific levels of ploidy generating particularly large serosal nuclei (e.g. [18,104]). In fact, cessation of mitosis and switch to the endocycle is among the earliest features of tissue differentiation in the serosa, and even in the *Drosophila* amnioserosa [24,129]. Many purported tissue-scale functions of polyploidy are probably applicable in this outer EE tissue. Serosal tissue integrity as a barrier epithelium of large cells confers cellular protection via detoxification [130] and innate immune responses to infection [55,56]. Furthermore, in many insect species, the serosa secretes a substantial cuticle that provides desiccation resistance [131–133] and mechanical protection [24]. Thus, polyploidy—and perhaps selective amplification—may support the serosa's capacity to transcribe numerous parallel copies of genes encoding key factors such as antimicrobial peptides and cuticle structural proteins. However, the genomic basis of serosal tissue properties awaits direct investigation. Ongoing developments in single-cell profiling will provide quantitative evidence on exact polyploid architectures, including tissue-specific copy number variants and the extent to which transcription scales with ploidy and locus copy number.

## 8. Ecological contexts and conclusion

EE tissues are physiological intermediaries as well as protective outer barriers. We noted degrees of EE tissue reduction in flies (§3), while marsupials only briefly require EE tissues before developing in a pouch (§7). Here, we address the wider ecological–developmental diversity seen across species (figure 1: ‘embryonic environments').

Although mammals are predominantly viviparous and sauropsids and insects are mostly oviparous, there are notable exceptions, with egg-laying monotremes and some viviparous insects. Viviparity is a particular form of matrotrophy, the provision of nutrition pre- or post-natally by the mother [134]. Postnatal parallels in insects and amniotes include honey bees' secretion of royal jelly to feed queen larvae and breast-feeding in mammals. Matrotrophy is also striking for the roles played by EE tissues. In amniotes, we touched on EE contributions to the placenta in the previous section, and further functions in mediating nutrition have been extensively reviewed (e.g. [135]).

Viviparity, known for less than 1% of insects, is predominantly restricted to three specialist lineages [134], and modifications of EE tissues in this context have thus far received limited but tantalizing study. Viviparity in aphids involves substantially smaller, yolkless eggs with rapid development in summer months, during the parthenogenetic phase of the life cycle, compared to overwintering oviparous eggs that retain a fully enclosing serosa that secretes a protective cuticle [136,137]. In the endoparasitic Strepsiptera, females often never emerge from the host, while in turn developing embryos surrounded by maternal tissue leave the ovary and move freely through the maternal hemolymph [20]. Third, the dipteran superfamily Hippoboscoidea, including tsetse flies (Glossinidae), provide nourishment in the uterus via specific gland-like structures, and this is underpinned by novel, lineage-specific milk proteins [138]. A few other instances of viviparity are also known. Developmental differences in eusocial termites and closely related cockroaches with parental care await further investigation [139,140]. Showcasing convergent similarities to placental development in mammals, earwigs (Dermaptera) develop a structure known as the pseudoplacenta, which is formed by the amnion and serosa together with the maternal follicular epithelium [20].

Oviparous insects also differ in their requirements for fully formed EE tissues. The apocritan Hymenoptera include parasitoid wasps, such as *Nasonia vitripennis*, which oviposit into the living tissues of a host (often another insect) and eusocial species with caste-based brood care in hives, such as the honey bee. These nutritionally rich and physiologically dynamic environments are associated with a reduced amnion that does not form an amniotic cavity, as well as—for parasitoids—polyembryony and post-hatching redeployment of serosal cells to modulate the host immune system (reviewed in [6]). However, classical histological analyses of sawflies, which lay their eggs externally on plant tissues, suggest that a reduced amnion may be a widespread trait within the Hymenoptera, irrespective of the embryonic environment ([141], and references therein).

Away from highly specialized, protected external environments, insect eggs exhibit diverse levels of terrestrial adaptation. *Drosophila* oviposits into humid, rotting fruit and eschews any EE covers, yet mosquitoes depend on serosal cuticle production to contend with transient aquatic environments [131], and many other insects are also aquatic. The ancient and speciose insects also present wider diversity in early amnion morphogenesis (beyond the modes depicted in figure 2), such as early serosal–germ rudiment disjunction in a few diverse lineages [6,23]. Given the high level of parental care and pervasive viviparity in amniotes, even with hundreds of millions of years of further evolution it seems unlikely that this animal group will reach an equivalent level of EE diversity.

That insects invented the amnion far before amniotes may surprise vertebrate researchers. But it is undeniable that although there are several functional and many genetic differences between the insect and the amniote amnion, there are also striking similarities. In this regard, *Tribolium*—combining complete amniotic cavity formation with an array of genetics research tools—can offer a suitable model to investigate certain aspects of early amnion development, offering a naturally *ex vivo*, accessible alternative to the amniotes. At the same time, the recent extended molecular knowledge of germ layers in vertebrate EE development, particularly from single-cell transcriptomics datasets, should provide a strong backbone for future research on EE genetic signatures in insect epithelia.

## Data Availability

This article has no additional data.
